# Conceptualising and assessing health system resilience to shocks: a cross-disciplinary view

**DOI:** 10.12688/wellcomeopenres.17834.1

**Published:** 2022-05-13

**Authors:** Sharif A. Ismail, Sadie Bell, Zaid Chalabi, Fouad M. Fouad, Reinhard Mechler, Andrada Tomoaia-Cotisel, Karl Blanchet, Josephine Borghi

**Affiliations:** 1 Department of Global Health and Development, London School of Hygiene & Tropical Medicine, London, WC1H 9SH, UK; 2 Institute for Environmental Design and Engineering, University College London, London, WC1E 6BT, UK; 3 Faculty of Health Sciences, American University of Beirut, Beirut, Lebanon; 4 Advanced Systems Analysis Program, International Institute for Applied Systems Analysis, Laxenburg, A-2361, Austria; 5 RAND Corporation, Santa Monica, 90401-3208, USA; 6 Department of Public Health, Environments & Society, London School of Hygiene & Tropical Medicine, London, WC1H 9SH, UK; 7 Geneva Centre of Humanitarian Studies, University of Geneva, Geneva, 1211, Switzerland

**Keywords:** Health system, shock, resilience, adaptation, transformation

## Abstract

Health systems worldwide face major challenges in anticipating, planning for and responding to shocks from infectious disease epidemics, armed conflict, climatic and other crises. Although the literature on health system resilience has grown substantially in recent years, major uncertainties remain concerning approaches to resilience conceptualisation and measurement. This narrative review revisits literatures from a range of fields outside health to identify lessons relevant to health systems. Four key insights emerge. Firstly, shocks can only be understood by clarifying how, where and over what timescale they interact with a system of interest, and the dynamic effects they produce within it. Shock effects are contingent on historical path-dependencies, and on the presence of factors or system pathways (e.g. financing models, health workforce capabilities or supply chain designs) that may amplify or dampen impact in unexpected ways. Secondly, shocks often produce cascading effects across multiple scales, whereas the focus of much of the health resilience literature has been on macro-level, national systems. In reality, health systems bring together interconnected sub-systems across sectors and geographies, with different components, behaviours and sometimes even objectives – all influencing how a system responds to a shock. Thirdly, transformability is an integral feature of resilient social systems: cross-scale interactions help explain how systems can show both resilience and transformational capability at the same time. We illustrate these first three findings by extending the socioecological concept of adaptive cycles in social systems to health, using the example of maternal and child health service delivery. Finally, we argue that dynamic modelling approaches, under-utilised in research on health system resilience to date, have significant promise for identification of shock-moderating or shock-amplifying pathways, for understanding effects at multiple levels and ultimately for building resilience.

## Introduction

Improving understanding of how health systems respond to shocks – such as infectious disease epidemics, armed conflicts or climatic events – has become a pressing issue. The West African Ebola outbreak in 2014–16, and more recently the coronavirus disease 2019 (COVID-19) pandemic, have spurred interest in health system preparedness, shock responses, and ways in which resilience to future events might be enhanced ^1– 3^. There is also a strong imperative for transformational change in health systems to bolster future resilience to climatic events given that the health sector is the fifth largest emitter of greenhouse gases globally and potential vulnerabilities to climate-related shocks are both substantial and rising ^4^.

Efforts to strengthen resilience must recognise that health systems are not simply the side-by-side collection of “all organizations, people and actions whose primary intent is to promote, restore or maintain health” as stated in the original World Health Organisation (WHO) formulation ^5^. Relationships between system elements at all levels from citizens to international organisations, and across sectors (beyond health) are fundamental to the functions that health systems perform and to achieving health outcome improvements ^5– 7^. Risk-based approaches that have been a mainstay of preparedness and response work to date assume some predictability in both shocks and system responses; they also prioritise stability and control within structures that are conceptualised in hierarchical terms ^8^. By contrast, social systems – including health systems – are complex. Interdependencies within and between them imply high levels of uncertainty in the response to future events, reducing the utility of probabilistic approaches for managing risk ^9^.

Resilience thinking offers approaches for better managing uncertainty ^10^. However, conceptualisations of resilience – and the nature of its relationship to other concepts such as vulnerability, fragility, responsiveness and sustainability – remain under development ^11– 16^, and recent reviews of the health literature have identified a series of limitations ^11, 17– 22^ including, firstly, a lack of consensus on definitions of resilience, and a tendency to consider, interchangeably, responses to very different kinds of shock. Clarifying the kind of shock a system faces is important not only in differentiating acute events from the chronic stressors (e.g. workforce shortages) facing many health systems worldwide, but also for understanding the scope of potential shock effects ^11^. Secondly, existing health systems research emphasises mechanisms for absorbing or adapting to shocks but rarely considers transformational change (i.e. wholesale structural change or goal re-orientation). Thirdly, and relatedly, existing research has engaged only to a very limited extent with health system learning and the contribution this can make to resilience. Finally, all studies and disciplines highlight a need for better tools for resilience assessment and measurement ^17, 18^ including enabling robust appraisal of health system performance and proper targeting and evaluation of resilience-bolstering interventions. Existing health system resilience assessment and measurement tools are index-based rather than dynamic, inconsistent in their choices of indicators and have not been widely evaluated ^20, 23^.

Over the past 20 years, valuable insights regarding resilience have emerged from disciplines, including environmental science, economics, industrial engineering, organisational theory, disaster studies and urban studies, which are relevant for health. This paper draws on recent literature on system shocks and resilience in these disciplines to guide health systems researchers interested in resilience and its measurement. The review considers several aspects of resilience: conceptualisation of shocks, definition and conceptualisation of resilience as well as attributes and behaviours of resilient systems, and approaches to assessment and measurement of resilience.

## Defining and conceptualising shocks

There is no consensus definition of system shocks in the literature on health system resilience, but two main areas of research can be identified. On the one hand are studies concerned with immediately recognisable shocks such as pandemics, natural disasters, national or international financial crises and armed conflict (e.g. 19). On the other hand, are studies concerned with the effects of chronic, largely internal stressors (e.g. workforce shortages, payment delays, or policy changes), drawing primarily on insights from local or regional health systems particularly in sub-Saharan Africa ^24, 25^.

Shock conceptualisation in other fields, particularly in socioecological systems (SES – an approach to conceptualising natural systems that links ecological and social or institutional subsystems to better explain the effects of resource management) and in economics, offers insights for health systems researchers in three main areas: [i] the need for clarity on shock intensity and scope, [ii] specifying the relationship between a shock and a system of interest, and [iii] the role of path dependencies in shaping shock effects.

**Figure 1.**
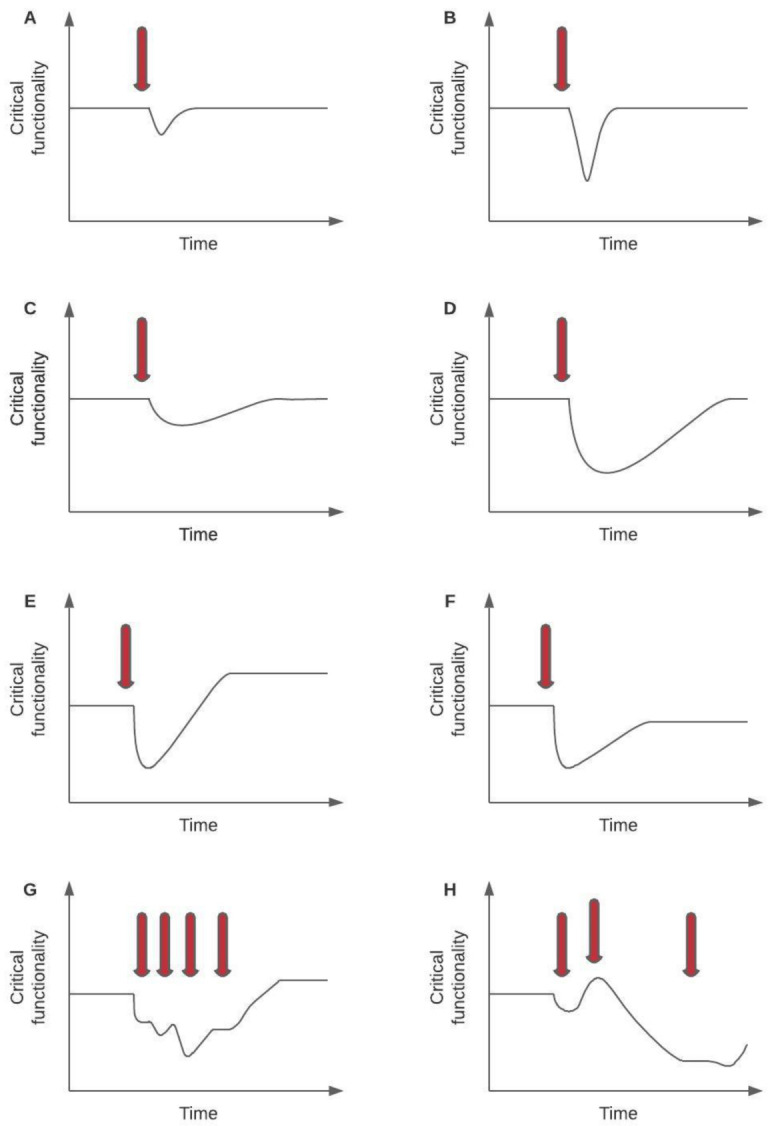
Relationship between the nature of a shock, prior resilience state of the system, and impact. “Critical functionality” here refers to a key outcome measure of interest that captures overall system performance. In panel A, a low magnitude disturbance hits a resilient system and both the impact and recovery trajectories are tight. In panel B, despite a high magnitude disturbance, a resilient system recovers quickly. In panel C, a low magnitude disturbance hits a low resilience system and recovery is sluggish. In panel D, a high amplitude disturbance hits a low resilience system with severe initial impact and recovery is sluggish. In some instances a disturbance of this size might be sufficient to cause transformation with either positive (panel E) or deleterious (panel F) effects on critical system functionality, however defined. Panels A-F all show varying effects of a single shock. Panels G and H illustrate potential trajectories in the face of sequential shocks of varying magnitude. In panel G, a series of low amplitude shocks progressively degrade system performance but recovery is achieved. In panel H, an initially low amplitude shock is followed by a much larger one from which recovery is limited (Figure adapted by permission from Springer Nature from Ref 9, © 2014).

Clarity is needed regarding shock intensity and scope to prevent conflation of truly acute events with long-term stressors or trends that may bring about very different dynamics within a system (potential trajectories following a shock, or series of shocks, are illustrated in Figure 1). In ecology, “disturbances” or “perturbations” encompass the disruptive effects of human activities and sudden climatic changes leading to rapid population loss, among other events ^26^, and close attention is paid to the duration and intensity of impact ^27^. Scale effects emerge strongly from work in economics, where the impact of both acute events and chronic stressors (e.g. financial crises or economic recessions, oil price shocks or fiscal policy changes due to geopolitical events) are considered but with an emphasis on effects at multiple geographical scales, especially on regional economies ^28– 30^.

Cross-scale effects are also important in work on “systems-of-systems” (i.e. systems whose elements are themselves sub-systems) in engineering, helping to explain how and why a shock may affect a given system as it does. In this view, component sub-systems are focused on discrete objectives or outcomes, but their activities and behaviours are complementary with other sub-systems within the whole system. For example, subsystems in health could be defined by function (e.g. medicines logistics and supply chain systems, or primary care service delivery systems), or by scale of operation (e.g. local, regional, national). Taken together, these systems-of-systems are capable of tasks that component sub-systems could not achieve individually, but also of generating emergent behaviours in the system as a whole, arising from the activities of the individual sub-systems ^31^. With respect to shocks, existing literature identifies a spectrum from massive shocks outside the boundaries of a system that disrupt all sub-systems within it, to localised disruptions that affect component sub-systems (and perhaps arise from within them) but not the whole ^31, 32^.

The question of whether a shock should be considered external (exogenous) or internal (endogenous) to the boundaries of the system is a recurrent theme across literatures. Even where shocks are considered exogenous, there may be important relationships with the system: the size of a disturbance influences risk to the system in the immediate term (alongside other factors such as prior preparedness and vulnerability); and overall effect and impact on recovery trajectory depends on underlying structures and processes. Some work in socio-ecology distinguishes between external drivers of change (the fact of the shock) and internal system variables that are vulnerable to them, and which in turn control the dynamics of other variables within the system. Walker *et al.*, for instance, contrast “fast” (variables of core concern in a system, that change rapidly) and “slow” (background variables that change much more slowly) system variables that can interact to produce quite different effects, amplifying some shocks, dampening others ^33^. Contemporary work on systems-of-systems considers both truly exogenous shocks (e.g. external to the system but affecting inputs, and with the potential to disrupt all intra-system elements as a result), and endogenous shocks originating from local disruptions or even emergent behaviours from within the system that have deleterious effects ^32^.

Finally, a key contention from studies on SES is that shock effects are path dependent ^34^. The nature and the scope of the response may depend on the phase of what is known as the “adaptive cycle” a system finds itself at the point a shock occurs. Adaptive cycle theory – applied not just to socioecological systems but to social systems more broadly – suggests that systems do not tend to a fixed and stable equilibrium point *per se* but instead move through phases over their life cycle, from rapid growth, through conservation to collapse (“creative destruction”) and finally renewal or re-organisation ^35^. The implication is that the impact of a shock is likely greater at specific points in the cycle when systems are unstable. Similar arguments regarding the importance of path dependencies emerge from wider research on the health effects of disruptive political and economic shocks, where studies suggest that pre-crisis exposure to health risk factors (alcohol, tobacco consumption etc), as well as the strength of social capital and the robustness of social protection systems are important in explaining differential health outcome trends arising from economic crises as diverse as the Great Depression in the 1930s and post-Communist transitions in Russia and Eastern Europe in the 1990s ^36^.

In summary, consideration of shock timing, scope and effects at different system levels are all likely to yield useful insights in terms of system elements and pathways influencing resilience. In particular, the prior state or configuration of a system matters, both in terms of vulnerabilities but also the presence of elements with the potential to amplify or potentially dampen shock effects. Finally, health system resilience assessment and measurement approaches may benefit from closer attention to the dynamic effects shocks produce within systems. 

## Defining and conceptualising system resilience to shocks

### Resilience definitions and attributes

Table 1 shows the diversity in conceptualisation of resilience across fields, from emphasising persistence and equilibration (e.g. in classical ecology and industrial engineering) through to adaptation, learning and self-organising capability (economics and socio-ecology). In the health systems literature as in work on SES and other social systems, however, there is some consensus regarding key characteristics of resilient systems. These include “hard” attributes such as availability of material and human resources, and the existence of collateral pathways (in health system terms, the existence of multiple mechanisms through which, for example, medical products or health services can be delivered) – echoed in some work in engineering ^32^. Effective information management is also vital in both engineering and social systems ^12, 32, 37, 38^, although there is less clarity on best approaches. For health systems, for example, the balance between formal surveillance and softer, more immediate data from human intelligence systems in shaping system responses has emerged as an area of debate in humanitarian settings and in the context of COVID-19 responses ^39, 40^.

**Table 1.** Definitions, terms and concept clusters linked to resilience in the literatures across research fields.

*Field*	*Summary definition*	*Conceptual focus(es)*	*Common, linked terms*	*References*
Health	Varied depending on the tradition (in other disciplines) on which studies draw	Predominantly macro-/meso-level focus on systems, but also a body of literature that addresses micro-/meso-level (organisational) perspectives. Predominantly normative interpretation of resilience, with some exceptions.	Adaptation, self- organisation, emergence (and clustered terms e.g. “collapse”?)	11, 17, 18
(Classical) ecology	System ability to withstand a disturbance while maintaining essential functions and relationships	Equilibration of systems and the speed with which it is possible to return to stable-state Magnitude of disturbances No explicit normative judgement on resilience	Resistance, buffer capacity, persistence, robustness	37, 44
Socio-ecology	System capacity to absorb disturbances and re-organise while undergoing change in order to maintain the same essential function, structure and feedbacks	Learning and innovation are central to resilience in SES systems Multiple equilibrium states are possible (even desirable) No explicit normative judgement on resilience	Adaptation, learning, innovation, transformability, tipping points	44, 45
Economics	Process by which economy withstands, adjusts and/or recovers from “market, competitive or environmental shocks”, if necessary by adaption, to return its prior developmental path or to move to a new, sustainable path	Often regional or local focus for investigation Interest in sustainability (or for purer macroeconomic studies, conventional markers such as GDP) as end-states for “developmental pathways” Dispute over the exogenous/endogenous nature of shocks	Vulnerability, recovery, robustness, adaptability	29, 30
Development studies	Development resilience as the capacity over time of a person, household or other aggregate unit to avoid and overcome stochastic poverty traps in the face of various stressors and in the wake of myriad shocks.	Predominantly micro-level agency but can be aggregated up to macro-level, and increasing emphasis on systemic factors affecting vulnerable to adverse development outcomes.	Adaptation, self- organisation, transformation, role of agency	46– 48
Industrial engineering	System ability to sustain disruptions without discontinuity in the system’s function (or with as rapid a restoration as is possible if discontinuity does occur)	System behaviour under “normal” conditions offers key insights on behaviour under duress A system has an identifiable, and single equilibrium state Normative interpretation of resilience (i.e. as a desirable state)	Reliability, restoration, (system) failure, safety, efficiency, controllability, (early) detection	12, 44
Organisational theory	Maintenance of positive adjustment under stress so that an organisation emerges strengthened and more resourceful	Distinction between organisational “software” and “hardware” in explaining resilience Normative interpretation of resilience (i.e. as a desirable state).	Self-organisation, planning, adaptation	49
Climate/disaster risk studies	System capability to resist, absorb, accommodate and recover from hazard events and trend after exposure, in a timely and efficient way plus increasingly capacity for transformation	Multi-level resilience (with a large body of literature focusing on micro- and community-level perspectives). Predominantly normative interpretation of resilience.	Anticipate, absorb, cope, recover, persist, transform (the latter largely in research, not yet in implementation), agency	14, 16, 46
Urban studies	Ability of a system to maintain or rapidly return to desired functions in the face of a disturbance, to adapt to change, and to quickly transform systems that limit current or future adaptive capacity	Multi-level focus linking micro- through to macro- perspectives Focus on temporal dynamics Normative interpretation of resilience (i.e. as a desirable state)	Sustainability, recovery, adaptability, re-organisation	44, 50

“Soft” attributes include networking and connectivity (or collaboration in human systems) ^26^, key determinants of shifts from one phase to another in the adaptive cycle in SES ^41^, and of how effectively an industrial engineering system-of-systems operates ^42^. However, trade-offs (e.g. between efficiency and adaptability in systems, between addressing immediate issues versus structural issues) are important, and networking between elements can undermine system resilience if the degree of connectivity is such that structures become too rigid to enable change ^41, 43^. In social systems, effective governance and leadership are important in maximising the potential of networking and connectivity between the actors of the system for resilience, but empirical studies of resilience governance and system leadership have been few including in health, although existing health research does identify attributes including legitimacy and knowledge management as important ^19, 37, 38^.

### Resilience as an outcome or as a process?

Resilience can be understood as a process or an outcome, or both. While much work in industrial engineering and socio-ecology, for example, measures resilience directly as an outcome (e.g. by trying to quantify the scale of disturbance a system can sustain and still return to a defined steady state), outcome-focused analyses of resilience in health systems are rare. This perhaps reflects unease about the normative implications of measuring resilience as an end in itself rather than changes in desired health outcomes, but also the intrinsic difficulty of identifying summary measures of resilience given the diversity of health systems functions ^11^. In this section, we argue that in the absence of broadly accepted summary measures for health system resilience, conceptualizing resilience as a process is likely to prove more fruitful for health systems researchers.

Existing health systems research distinguishes three processes contributing to resilience, drawing on the literature from socioecology: [i] absorption (no structural change occurs; the shock is simply accommodated using existing structures and pathways – sometimes referred to as persistence in the urban studies literature); [ii] adaptation (where certain, circumscribed structural or pathway changes occur to respond to the shock); and [iii] transformation (in which learning is harnessed to fundamentally alter the structure of the system and strengthen it for the future, with or without goal re-orientation) ^19, 49^. The focus in empirical health research has predominantly been on absorption and adaptation, ranging from studies of effects of shocks on critical health outcomes or utilisation of essential services through to changes in service delivery models and coping strategies of health care workers ^17, 51– 53^. Studies of transformational change in health systems are largely absent, a deficit perhaps linked to a misperception that resilience theory prioritises stability over the pursuit of just and equitable health outcomes for populations ^17, 54^. Finally, none of this work explicitly links these processes in a conceptually coherent theory of how and why system resilience arises.

Thinking on adaptive cycles in social systems, however, emphasises system learning, adaptation and reorganization, and positions transformability (i.e. the capability to create new structures or reorient system goals when conditions make continuation of existing arrangements impossible) as a fundamental attribute of resilient systems ^55^. In its original formulation, the adaptive cycle incorporated four stages – system growth, equilibrium, collapse and reorientation – in a feedback loop. Equilibrium was achieved when the maximum potential of a socioecological system was reached, and with the highest level of connectedness between system elements ^56^. Recent adjustments to this formulation for application to social systems have recognized that connectedness can, *in extremis*, undermine the durability of a system by reducing flexibility to changing circumstances, but have also emphasized that, if we consider a specific scale of analysis, a system operating at that scale is subject to pressures from elsewhere that may amplify or constrain the potential for change ^57, 58^. An appreciation for multi-scale dynamics and cross-linkages – or “panarchy” – is therefore fundamental to understanding how a system can be both resilient and concurrently show transformational capability ^45, 57– 60^.

**Figure 2.**
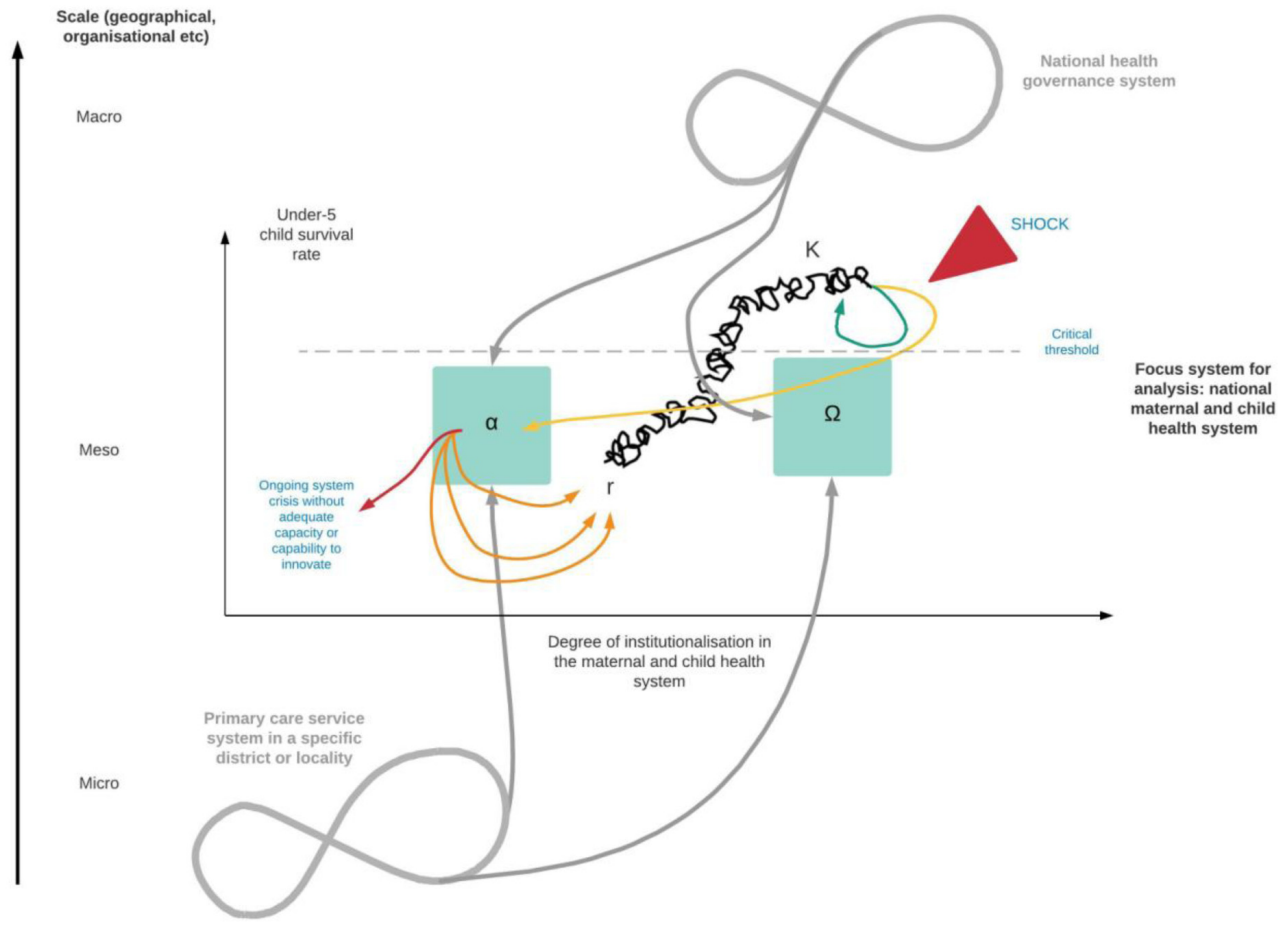
Visual representation of the adaptive cycle applied to a national maternal and child health system. Looking at the focus system in the centre of the diagram, there is steady but uneven improvement in the under-5 child survival rate as system processes and behaviours become progressively more institutionalized, towards an equilibrium point (K). A crisis or shock (red triangle) can be absorbed provided its effects are within a certain threshold, and in this case the system rapidly returns to the general upward trajectory in child survival (green line). If the shock impact exceeds a critical threshold, the system will move towards a zone of instability (Ω) during which structures and processes are disrupted (yellow line). A period of innovation (α) may give rise to system renewal, restructuring or reorientation and return to an upward trajectory (r), but pathways to this can be variable (orange lines). If no innovation occurs, further decline may ensue leading potentially to irrevocable declines in child survival (red line). The key points of vulnerability – and conversely maximal opportunities for change – are α and Ω; these are also the critical interaction points at which processes in linked systems (in this case the national health governance system at macro-level, and primary care service delivery system at micro-level) at other scales could push the maternal and child health system towards transformation (Figure adapted from reference number 57; the original figure on which it draws is licensed under CC BY-NC 4.0).

Figure 2 applies this thinking to a health case study: the maternal and child health system in any given country, chosen because this is frequently among the first domains in which health service use and population health outcomes begin to be affected in the event of a shock ^61– 65^. We can think of the maternal and child health system as bringing together population demand and service supply dynamics. In this visualization, the system enters a period of growth (point *r*) at the initiation point of a new form of structural organization (e.g. due to introduction of systemic reform). There is then an upward trajectory as changes become progressively more institutionalized, population reach increases and service performance (in this case proxied by improvement in the under 5 child survival rate rate) improves. As the figure shows, however, this trajectory is rarely linear and there may be periodic setbacks followed by periods of improvement (black scribble line). At the point of maximal institutionalization of the reformed system structure ( *K*), further growth potential tails off and improvements in under 5 child survival begin to stagnate.

Periodic shocks of varying magnitude may occur at any point along this pathway but, provided they are small enough, these are managed by the system using existing reserves and processes (e.g. surge service capacity, or redeployment of healthcare workers to areas where demand is highest), so that there is a rapid return to the overall upward trajectory for child survival (green line). Beyond a certain threshold, however (grey dashed line), the existing system structure begins to become unstable and rules or pathways governing predominant modes of service delivery begin to break down (yellow line through Ω to α). In systems with sufficient capacity and capability to innovate, there may be a period of rapid testing of alternative approaches, such as new service delivery models or task-shifting for healthcare workers (α), leading – if successful – to a new period of outcome improvement based on a renewed or reorganized system structure and associated behaviours. Pathways to this renewal or reorganisation can be variable, however, with some leading to marked initial declines in child survival (orange lines). Without this, however, further declines in institutional capability to deliver services risk progressive or even sudden declines in under-5 mortality over time (red line).

How a system behaves across this cycle is determined at least in part by pressures from the larger-scale and smaller-scale systems with which it interacts (e.g. geographically or hierarchically) – and this is where the potential for transformative change arises. In work in socio-ecology, environmental science and urban studies, transformation has been conceptualised either as changes in mechanism (e.g. completely new activities, shift in geographical scale or intensity of an existing activity, or a values-based reorientation in delivery), or changes in system objectives over time and space ^66, 67^. In the modified adaptive cycle in Figure 2, changes in system structure and behaviour arising from within the system alone are likely to be absorptive or at most adaptive; transformative change will typically require resources, or a “push”, from outside the maternal and child health system. These cross-scale interactions have the greatest potential for impact – either positive or negative – at points α and Ω – when the system is unstable. If the shock is national in scope, then there may be critical losses in, for example, health financing or leadership capacity nationally that accentuate the overall impact of the crisis at point Ω on the maternal and child health system. There may also be destabilizing effects at the micro-level, such as loss of income for service users, that reduce service uptake and the likelihood of sustained improvements in under-5 child survival returning. At point α, on the other hand, innovations from linked but smaller-scale systems (e.g. new local-level outreach service models) or macro-scale systems (e.g. mobilization of emergency funding to support service delivery nationally, changes in leadership or governance reforms) may combine in new ways to enable transformative change within the maternal and child health system ^57, 68^. 

What specifically prompts transformational change? There is a large body of evidence (e.g. from development studies) demonstrating that shocks can act as spurs to transformative, and positive, change for better long-term outcomes ^14^. In socioecological and environmental systems, transformations may occur through the combined effect of external pull factors (e.g. to respond to a demonstrable need outside the system) and within-system forces – which may be top-down (e.g. active management) or bottom-up (e.g. collective action) – overcoming opposing forces ^58, 69^. Crucially, resilience is necessary for a transformational path to be maintained once change in this direction begins to occur ^69^. Transformations need not have positive effects: there is a large literature in ecology on deleterious, even catastrophic ecosystem transformations due to disruption, for example ^70^. The extent to which a change can be seen as transformative also depends on the scale of analysis. A transformative change in service delivery models at a local level, for instance, is unlikely to materially affect the macro-level structure of a system, though local effects can be substantial ^45^.

There are three main implications from this work for health systems researchers. First, focusing on resilience processes rather than outcomes liberates resilience thinking from at least some of the normative constraints for which it has been criticised, and offers alternative avenues for assessment and measurement in the absence of broadly agreed metrics or indices for health system resilience. Second, research designs that adopt multi-level perspectives on system responses are much more likely to yield meaningful insights on the sources of resilience (or otherwise) to shocks, and to identify transformational changes that may occur even in the absence of whole-system reconfiguration. Finally, both of these insights suggest that dynamic approaches that incorporate feedback are likely to be central for future work on operationalizing resilience – the subject of the next section.

## Assessing and measuring system resilience

**Figure 3.**
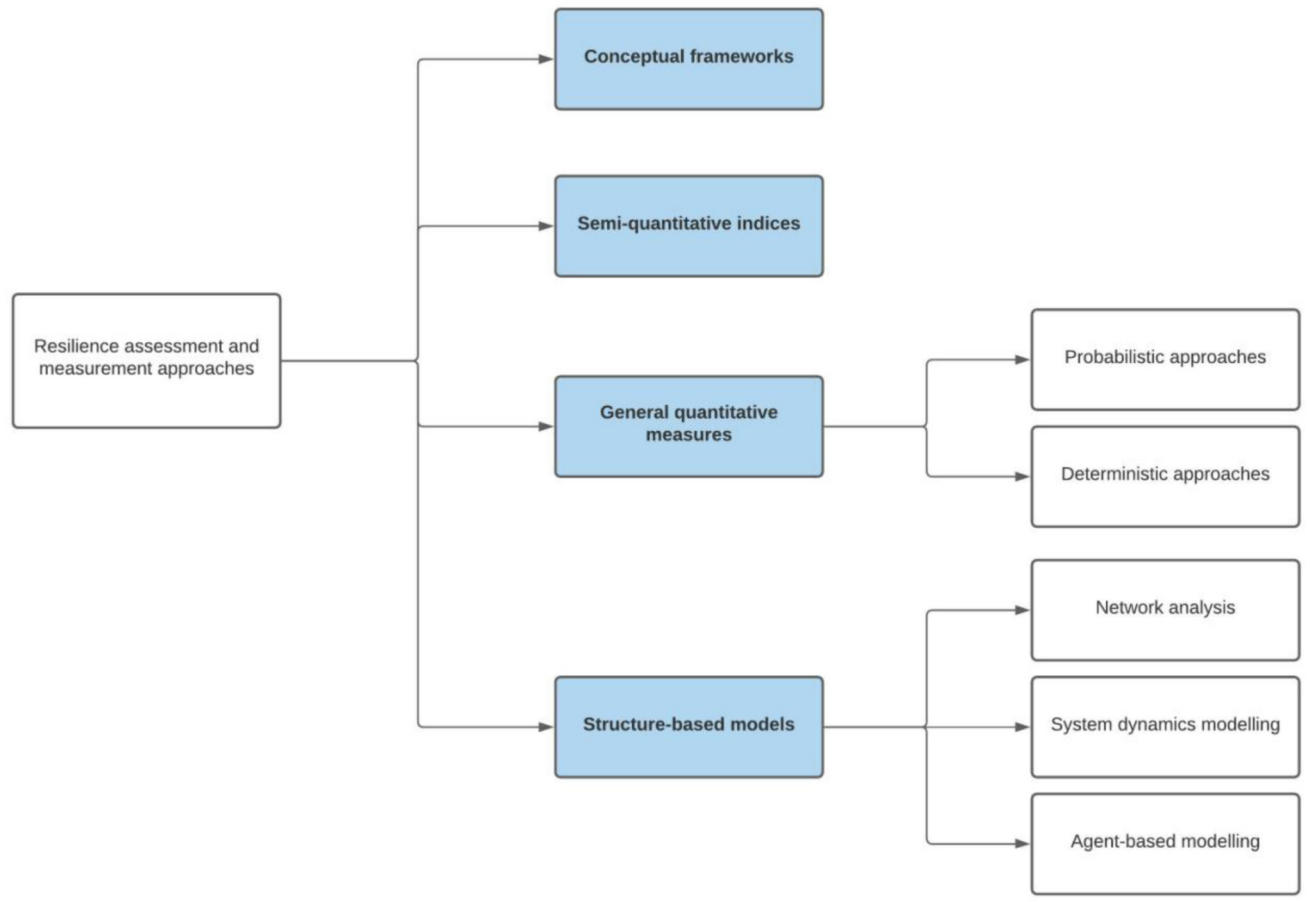
Simple classification of approach to resilience assessment and measurement. The four broad approaches to assessment and/or measurement considered in detail in the main text are highlighted in blue below, with specific methodologies highlighted in the right hand column (Adapted from Ref 12, with permission from Elsevier).

Operationalisation is a particularly challenging area of resilience research. It is helpful to distinguish assessment, which is intended to inform management interventions principally by identifying risks, opportunities and alternative strategies to change (sometimes as a precursor to purposeful transformation); and measurement, which is concerned with early detection of change for situational awareness purposes ^44, 71^. Four main approaches to operationalisation can be identified in the literature (highlighted in blue in the summary Figure 3): the use of [i] qualitative conceptual frameworks; [ii] semi-quantitative indices or metrics of resilience; [iii] conventional quantitative (statistical) approaches; and [iv] systems modelling. In this section, we describe and critically assess the potential of each of these approaches for quantification of health system resilience. Drawing on the material on adaptive cycles presented above, we argue that dynamic modelling approaches are likely to offer the greatest benefit for health systems researchers in future.

### Methodological approaches in health systems research

Qualitative approaches – in which conceptual frameworks are used to guide investigation of resilience – are by far the most common in health systems research, but even in the empirical literature, few studies use frameworks to guide their analyses ^17^. Those that do typically reference either [i] a framework inspired by insights from ecology, focused on resilience governance ^38^; [ii] frameworks linked to the WHO building blocks which consider dimensions of resilience at whole-system level ^20, 72^; or [iii] a framework drawing on organisational theory, but emphasising absorptive, adaptive and transformative approaches to managing chronic stressors ^73^. Variants of these approaches exist but with few applications in health so far ^74^, although there is some work applying the absorb-adapt-transform triumvirate to resilience ^75, 76^.

A second strand of health systems research uses semi-quantitative indices in which existing process and outcome measures are repurposed as proxies for resilience ^72^. Recent work by WHO Europe repurposes common health system metrics (e.g. level and geographical distribution of health sector spending; health worker remuneration and absenteeism; the existence of contingency plans) to build a picture of health system reserve and capacity to respond to shocks that enables iterative monitoring ^23^. Indicator-based approaches have also been applied to assessment of vulnerability and health system resilience to climatic events, including with a view to *ex ante* “stress-testing” ^77– 79^. These approaches have advantages of simplicity and analytic familiarity but are limited by a high level of abstraction that reduces scope for empirical application (for conceptual frameworks), and because they do not capture dynamic behaviour. The primary means for gathering information on changing system behaviours over time is through iterative indicator assessment, but static frameworks offer no information on pathways affecting these behaviours or on cross-scale effects influencing them ^44^.

### General quantitative measures used in other fields

General quantitative measures have been used in socio-ecology, environmental science and industrial engineering to directly measure resilience attributes such as robustness or elasticity, but have not to date been applied in health systems research ^12, 44^. General measures have been applied – for example – to assessment of infrastructure resilience (e.g. transport), to measure system robustness, rapidity of recovery after a shock and redundancy among other characteristics. These measures provide an indication of how a system would respond to a shock but do not account for the probability of that shock occurring in the first place. For this, probabilistic approaches are preferred and have been used to evaluate infrastructure responses in the event of earthquakes, for example ^12^. In SES, quantitative assessment of skewness or increasing variance from stable points for system critical variables can provide early signs of an impending transformation from one stable equilibrium to another as a disturbance pushes a system closer towards a threshold point, as can critical slowing down (CSD) i.e. increasing delays in system recovery from disturbances as a threshold point is reached ^71, 80^. However, quantitative measures are not always reliable indicators of change, a drawback of metrics-based approaches that has long been recognised in the wider health systems literature ^81^. The crudity of general measures also means the risks of false alarms can be high, or conversely that other behaviours signalling an impending change are missed; research in SES shows that a sudden, catastrophic shock may, for example, result in transformation without a preceding slowing down ^80^. Finally, these approaches give little insight into system structure, or dynamic behaviours. Integrative approaches to resilience assessment and measurement that combine quantitative indicator use with mapping and other methods may be better for identifying system attributes contributing to resilience over time ^80^, but these too do not capture networking or dynamic behaviour.

### Modelling approaches

Structural modelling approaches provide methods not only for delineating system structure and linkages across levels, but also for identifying leverage points for action that may contribute to preserving, sustaining, and strengthening resilience. They can also be used in a wide variety of ways, from theory generation in terms of the kinds of principles and processes governing system behaviour, through to structurally realistic models that to inform policy or management of social systems ^82^. Here, we consider three mixed-methods approaches for exploring system resilience: network analysis, System Dynamics Modelling (SDM) and Agent-Based Modelling (ABM).

Network analysis can help identify components of networks with critical effects on system resilience and linkages between these. There is some – albeit limited – track record of applying these approaches to health system questions. Empirical work looking at the effect of a system shock on managerial decisions in a regional eye health system in Ghana, for example, showed that transformational changes in network organisation reduced overall resilience while simultaneously improving responsiveness to the needs of particular stakeholders in the system – emphasising that resilience is subjectively experienced, and that outcome selection matters ^83^. Computational modelling of national-level primary healthcare network resilience in Austria showed that following a shock (in this case resulting in large and sudden reductions in the size of the physician workforce) there can be distinct thresholds beyond which the ability of providers to maintain coverage of essential services is compromised ^84^. Elsewhere, recent work in engineering has considered the amplifying effects of connections between multiple, related networks (“network-of-networks”) when faced with shocks, especially where there are many feedback and feed-forward connections. This work highlights the need to identify and protect, or grant greater autonomy to, critical nodes that can shape network responses to shocks as a means of bolstering resilience ^42^. However, network analysis has limited capacity for modelling dynamic behaviour, for which approaches such as SDM and ABM are more appropriate.

System dynamics has been defined as “the use of informal maps and formal models with computer simulation to uncover and understand endogenous sources of system behaviour” ^85^, and has been applied in a number of empirical studies on resilience in health. Typically, SDM involves production of a qualitative representation of the dynamics contributing to a problem of interest (a causal loop diagram or CLD) with an emphasis on feedback processes, which may then be translated into a simulation model with stocks and flows. The few existing SDM studies on health system resilience have predominantly used CLDs to represent system structure (including important variables, system boundaries and feedback loops), and have highlighted the importance of decentralised decision-making, institutional learning, accurate and timely information flows, and path dependencies in determining the extent to which systems have been able to respond to shocks ^75, 76, 86^. Applications of SDM in other fields have focused on participatory engagement in policy development to support system resilience, and quantification of resilience metrics such as robustness, time to recovery from shocks and elasticity, although broader insights from these works are obscured by varying resilience definitions among other limitations ^87, 88^.

ABM focuses on how agency between diverse individuals or institutions, as agents, can give rise to dynamic behaviours. It offers a promising avenue because of its ability to address effects related to network structure and heterogeneity between agents, although there are important trade-offs to consider in terms of time and resource intensity (including computational power) for modelling using ABM by comparison with SDM, which typically models at aggregate rather than individual level ^89^. In practical terms, applications to both engineering ^90^ and social systems including to SES have so far been few ^91– 93^. One study applying ABM to analysis of health system resilience was identified for this review, considering local-level service responses to earthquakes in the United States, and more specifically interdependencies between services including health that might affect response effectiveness ^94^. This study demonstrated significant interdependencies between health service and education providers at local level, suggesting that the integrity of social service provision can be highly vulnerable even to relatively small disruptions. 

A particular attraction of SDM and ABM is their flexibility in use, on the one hand, for hypothesis testing and exploration of new modes of working, and on the other in being linked to a greater or lesser degree to empirical data to inform real-world governance and management approaches ^82^. In reality, both resilience assessment and measurement are likely necessary to give a rounded sense of system resilience spanning structure and dynamics ^44, 80^. Modelling approaches by definition offer heuristics for system behaviour overall, and outputs should be interpreted with reference to empirical or observational research, over and above validation approaches commonly integral to the model development process ^91^.

## Conclusions

What can health system researchers investigating system resilience learn from advances in other fields? Two major domains of resilience research offer the most relevant insights: (i) work on social systems particularly in development, disaster, environmental and urban studies; and (ii) emerging thinking on system-of-systems in engineering. Work in both domains emphasises the importance of cross-scale interactions and transformability – both largely neglected topics in health systems research.

Four general lessons can be drawn from the literature considered here. Firstly, research on resilience should clearly describe shock characteristics such as intensity, duration, geographical scope, and try to identify system elements amplifying or conversely dampening shock effects. Without this, it is very difficult to properly interrogate shock effects.

Secondly, most empirical research on resilience in health continues to use flat perspectives on health system structure (in particular, the building blocks approach) addressing single levels of analysis and taking a uniform view on where system boundaries lie (principally, national boundaries). This framing is problematic for analysis of shocks such as climatic events or armed conflicts that can produce cascading effects at multiple health system levels within and across jurisdictions. The system-of-systems and network-of-networks approaches allow us to frame shock responses very differently, recognising that health systems combine interconnected sub-systems (both sectoral and across geographies) with different elements, behaviours and even objectives. Medicine supply chain sub-systems, for example, likely operate in fundamentally different ways from health governance and accountability sub-systems. As part of the reckoning with the impact of COVID-19, however, there is also growing recognition of the need to consider connectedness with broader societal systems, including economic, environmental and social ones, in bolstering health system resilience to future shocks of global scale ^7^. The methodological corollary of these observations is the need for mixed-methods research designs that interrogate resilience at multiple levels and across sectors.

Third, transformability should be considered an integral (rather than incompatible) property of resilient systems. Recent theoretical work applying the concept of an adaptive cycle in social systems reinforces the importance of links between systems across scales, and also provides a new way of thinking about how and when transformational change arises that can, as we have seen, be readily applied to health systems ^57, 58, 60^. Work on SES and urban systems also emphasises that, once a crucial tipping point has been reached and transformational change towards a new equilibrium state begins, resilience is a prerequisite for momentum to be maintained and transformation to realised.

Finally, although approaches to assessment and quantification of health system resilience remain nascent, modelling approaches including network analysis, SDM and ABM have significant potential to advance research in this domain. These three approaches have been applied to a very limited extent in health but can provide valuable insights on critical system elements through which shock effects are most likely mediated. SDM and ABM also capture dynamic behaviour in a way that conventional quantitative approaches cannot, to identify processes underpinning resilience and potential leverage points for intervention. Health systems researchers have an opportunity to lead the advancement of resilience research across fields, through application of these methods.

## Data availability

No data are associated with this article.
