# Supplementary material for: Conceptualising and assessing health system resilience to shocks: a cross-disciplinary view
Source: Wellcome Open Res. 2022 May 13;7:151. [Version 1] doi: 10.12688/wellcomeopenres.17834.1 (PMC11140310; doi:10.12688/wellcomeopenres.17834.1)
Supplement: Peer review history

# Reviewer report: Reviewer response for version 1

**DOI:** 10.21956/wellcomeopenres.19746.r82497

**Published:** 2024-05-30

**Authors:** Revathi N. Krishna

**Affiliations:** 1 Public Health and Preventive Medicine, Monash University, Clayton, Victoria, Australia

This paper attempts an in-depth analysis of 'resilience of the health system'. This is certainly a field that is of growing importance and need for more research. Given the current climate change, conflict impacting various parts of the world and how human movement is being conceptualized, it is imperative that strengthening the health system is key to ensure health care access and health equity. 

 The authors attempt to summarize the literature from across disciplines on conceptualizing 'shock', 'understanding the impact of shocks on the health system adequately, especially in the context of compounding and cascading impacts', 'resilience' and how one 'measures resilience of the health system'. The authors attempt to understand these key concepts using various disciplines including but environmental science, economics, industrial engineering, organizational theory, disaster studies and urban studies. 

 The authors acknowledge the complexity in defining and measuring resilience - whether it is an outcome or process. This has been an ongoing and enduring debate in the field of disaster studies, and public health with not much of a resolution and it is to the authors' credit that they delve into it using maternal and child health as an example of the health system.  Shocks and resilience, as the authors recognise are complex and multifaceted. Exploration of examples of shocks and resilience in the literature would be useful. 

 It would be helpful and replicable if the authors provide a detailed methods section in this review paper. This would make research replicable, thus strengthening this important work. 

 Some parts of the paper are quite dense and while it is a good read, it might exclude many readers. Perhaps there is value in re-reading this from a accessibility of communication perspective. 

 I agree with the other reviewer that this paper doesn't delve deep enough on health policy and systems research. Perhaps, their methodology would explain why they weren't able to tap into that literature. 

 A cross-disciplinary review such as this is valuable in the current world where cross-disciplinary work is rising and necessary to do innovative scientific work.

Is the review written in accessible language?

Partly

Are all factual statements correct and adequately supported by citations?

Partly

Are the conclusions drawn appropriate in the context of the current research literature?

Yes

Is the topic of the review discussed comprehensively in the context of the current literature?

Yes

Reviewer Expertise:

Disaster Resilience,  Mental health in the context of trauma, specifically disasters.

I confirm that I have read this submission and believe that I have an appropriate level of expertise to confirm that it is of an acceptable scientific standard, however I have significant reservations, as outlined above.

# Reviewer report: Reviewer response for version 1

**DOI:** 10.21956/wellcomeopenres.19746.r72920

**Published:** 2024-05-22

**Authors:** Zeyneb Foroughi

**Affiliations:** 1 Education Development Center, Iran University of Medical Sciences, Tehran, Iran

This study is valuable as it offers a unique perspective on assessing health system resilience. It can serve as a foundation for further practical research in this area. However, I have some comments based on a review I recently conducted in this area but these are not mandatory:

 Under the topic of "resilience as an outcome or as a process":

 1. It should be considered that considering resilience as outcome undermine it as the attribute of complex adaptive system and synonym with performance improvement.

 2. Regarding resilience as a process, you considered absorption, adaptation, and transformation as three seperated processes to achieve resilience. However, these are three complementary steps that work in relation to each other.

Is the review written in accessible language?

Yes

Are all factual statements correct and adequately supported by citations?

Yes

Are the conclusions drawn appropriate in the context of the current research literature?

Yes

Is the topic of the review discussed comprehensively in the context of the current literature?

Yes

Reviewer Expertise:

I am an assistant professor of Health Policy. I have worked on a 3-year research project on health system resilience during economic crises, two articles of it are currently published.

I confirm that I have read this submission and believe that I have an appropriate level of expertise to confirm that it is of an acceptable scientific standard.

# Reviewer report: Reviewer response for version 1

**DOI:** 10.21956/wellcomeopenres.19746.r53366

**Published:** 2023-01-20

**Authors:** Christina Zarowsky

**Affiliations:** 1 École de Santé Publique de l'Université de Montréal (ESPUM), Montreal, QC, Canada; 2 School of Public Health, University of the Western Cape, Bellville, South Africa

This important and interesting paper brings insights about the notion of “resilience” from a range of fields and disciplines outside health policy and systems research into the discussions within HPSR about how to conceptualise and measure the resilience of health systems to shocks. The authors present their synthesis and recommendations from fields outside of health in terms of 4 key issues: (1) The importance of clearly describing shock characteristics and identifying how system elements may amplify or dampen shock effects; (2) shifting from a flat and jurisdictionally-bounded perspective on health system structure to “system-of-systems” and “network-of-networks” approaches that recognize the complex and dynamic structures and relationships of sub-systems – this enables a dynamic analysis which can address the cascading effects of shocks (such as climate crises or conflicts) at multiple levels and both within and across jurisdictions; (3) the importance and value of fully integrating transformability into thinking, research and practice on resilience – to go beyond a tendency to focus on survival/coping and returning to a status quo ante; (4) approaches to measuring and evaluating health system resilience that can capture the complexity outlined above – the authors argue that modelling approaches such as System Dynamics Modelling (SDM) and Actor-based Modelling (ABM) are particularly promising for taking the field of resilience research further.

 This rich and very interesting paper is a pleasure to read and re-read. It offers a wealth of insights in considerable detail and promises to be an important resource for researchers in the field of health system resilience. The examples, the overall structure, and the conclusion are excellent.  I have no major critiques or recommendations to make, but there are a number of minor revisions that could strengthen the paper and make it more accessible to a range of readers. As it stands, it is a valuable resource for those already working in the field, but it is a bit too dense for a broader readership. It covers a tremendous amount of ground and could of course be several separate manuscripts. I believe that it is important to have comprehensive syntheses such as this one, which go into detail and address a range of issues, but it does make it challenging to follow at times.

 Suggestions:

1. A short “Methods” section would be very helpful.
2. Table 1 is very good, but it would be helpful to include some specific citations/references in the text when presenting insights from other fields. This might simply mean repeating the references from Table 1 when e.g. SES or engineering is mentioned, particularly when the authors make field-specific assertions such as “[s]hock conceptualisation in other fields, particularly in socioecological systems (SES – an approach to conceptualizing natural systems that links ecological and social or institutional subsystems to better explain the effects of resource management) and in economics, offers insights for health systems researchers…” or “Cross-scale effects are also important in work on “systems-of-systems” ….in engineering”. This would allow the reader to link insights to fields to references while reading the text (especially for those readers, like myself, who prefer narrative to tables).
3. Throughout the paper, sections and also paragraphs cover many topics which are related. This again makes for an excellent resource, but at times a tough read. It would be helpful to include a few more subheadings, for example for each of the 3 subsections of the “Defining and conceptualizing shocks” section. This may simply mean highlighting sentences such as “Clarity is needed regarding shock intensity and scope” at the beginning of the third paragraph of that section, “Cross-scale effects are also important” in the next paragraph, and “Finally, a key contention from studies on SES is that shock effects are path dependent” on the next page.
  Similarly, Figure 2 and the accompanying text are excellent, but I wonder if it is possible to have words instead of “alpha”, “omega”, K, etc., particularly in the text. Again, mathematically-oriented readers may have no trouble at all with the Greek but others may benefit from substantive words or short phrases.
4. In the section on transformational change, there is a tantalizing sentence which is referenced but begs one more sentence of explanation: “Crucially, resilience is necessary for a transformational path to be maintained once change in this direction begins to occur”.
5. The concluding paragraph of the conceptualization section mentions “normative constraints for which it has been criticized”. By whom? Perhaps just a reference.
6. The final section, on assessing and measuring system resilience, is an excellent overview of the 4 methodological approaches, but it feels a bit disconnected from the rest of the paper.
  Consider a) a concluding paragraph similar to those in prior sections, b) referring back quite specifically to some of the points/questions/challenges/ideas you have raised in previous sections.
  Finally on this section, it is not clear what exactly the authors mean when they state that dynamic modelling approaches "are likely to offer the greatest benefit for health systems researchers in future" - to do what, why, etc.? This would be a good point to reiterate, or specify a bit more precisely, the purpose and audience for this paper and especially for this methodological section.
7. Finally, I found that overall the paper did not quite convey the breadth and depth of work already done and being done in health policy and systems research. It comes across a bit as though no-one (or very few) in HPSR has thought about issues of complexity, dynamics, transformation etc, while in fact many of the cited authors – including co-authors of this paper – have been thinking and writing about these issues for some time and with both rigour and nuance. If the point is that these are the exception and the centre of gravity, as it were, is not there yet, that could perhaps be indicated. Or, perhaps it could be helpful to indicate that these insights from other fields help deepen and operationalise insights which are less developed (though not absent) in HPSR/health resilience research. This is how I read the paper and why I enjoyed it so much - there are many “Yes! This is really helpful” moments. Perhaps I am just feeling a bit defensive on behalf of these scholars, but I would not want readers not familiar with the work in the health resilience field to think that all of these insights are entirely novel and non-existent in HPSR. (This does NOT mean writing a lot more about the health literature – it is really an issue of one or two sentences or even phrases, I believe).

Is the review written in accessible language?

Partly

Are all factual statements correct and adequately supported by citations?

Partly

Are the conclusions drawn appropriate in the context of the current research literature?

Yes

Is the topic of the review discussed comprehensively in the context of the current literature?

Yes

Reviewer Expertise:

Global health; medical anthropology; health policy and systems; social determinants of health; vulnerability and resilience in conflict-affected areas; governance and equity in health

I confirm that I have read this submission and believe that I have an appropriate level of expertise to confirm that it is of an acceptable scientific standard, however I have significant reservations, as outlined above.
